# Sub-microscopic schistosomiasis and soil-transmitted helminths in school children: molecular diagnostic evidence and implications for disease elimination

**DOI:** 10.1038/s41598-026-44877-8

**Published:** 2026-03-18

**Authors:** Peter Asaga Mac, Keswet Darlington Anderson, Danaan Anthony Dakul

**Affiliations:** 1 Institute of Infection Prevention Control and Hospital Epidemiology, Universitätsklinikum Freiburg, 79016 Breisacher Straße 115b, Freiburg, Germany; 2 College of Health Technology Zawan, Jos, Plateau State Nigeria; 3 Department of Zoology, University of Jos, Jos, Nigeria

**Keywords:** Sub-microscopic infections, Molecular diagnostics, Multiplex real-time PCR, qPCR, Schistosomiasis, Soil-transmitted helminths, Diagnostic comparison, Nigeria, Diseases, Medical research, Microbiology

## Abstract

Schistosomiasis and soil-transmitted helminthiasis elimination programmes depend heavily on microscopic diagnostics, yet these methods may fail to detect low-intensity infections that sustain transmission. Molecular techniques could reveal the extent of submicroscopic infections missed by conventional approaches. To compare the detection rates of conventional microscopy with probe-based multiplex real-time PCR and quantitative real-time PCR (qPCR) for schistosomiasis and soil-transmitted helminths among primary school children in Plateau State, Nigeria, and to estimate the proportion of submicroscopic infections undetected by standard methods. In this cross-sectional study, 1,368 systematically sampled primary school children from six schools across six Local Government Areas provided urine and stool specimens. These were analysed using standard microscopy (urine filtration and Kato-Katz). A stratified subset of 585 samples (42.8%) underwent molecular analysis by probe-based multiplex real-time PCR for species detection and probe-based singleplex qPCR for quantification. Microscopy revealed an overall parasitic infection prevalence of 20.7% (95% CI: 18.6–22.9%). Among the 585 molecularly analysed samples, molecular methods detected 178 positive cases compared with 123 by microscopy, representing a 44.7% increase (95% CI: 36.2–53.8%, p < 0.001). An additional 9.4% of specimens (95% CI: 7.1–12.1%) were molecular-positive but microscopy-negative. For schistosomiasis, molecular methods identified 52 cases versus 36 by microscopy (44.4% increase), whilst soil-transmitted helminths showed 126 versus 87 molecular- and microscopy-positive cases respectively (44.8% increase). Molecular diagnostics identified 44.7% more infections than microscopy alone (95% CI: 36.2–53.8%, p < 0.001), indicating that conventional methods failed to detect a substantial proportion of low-intensity infections. These findings suggest that microscopy-based surveillance may underestimate infection prevalence in elimination programme settings. Integrating molecular methods into surveillance frameworks could improve the accuracy of elimination progress assessments.

**Supplementary Information:**

The online version contains supplementary material available at 10.1038/s41598-026-44877-8.

## Introduction

Schistosomiasis and soil-transmitted helminthiasis are among the most widespread parasitic infections worldwide, collectively affecting well over a billion people and constituting a major share of the neglected tropical disease burden^1^. The World Health Organization estimates that schistosomiasis caused principally by *Schistosoma mansoni*,* S. haematobium*,* and S. japonicum* affects approximately 230 million individuals, whilst soil-transmitted helminths, including *Ascaris lumbricoides*,* Trichuris trichiura*,* Necator americanus*, and *Ancylostoma duodenale*, infect over 1.4 billion^2^. Sub-Saharan Africa bears a disproportionate share of this burden, harbouring more than 90% of schistosomiasis cases and a substantial proportion of STH infections^3^.

These infections overlap geographically, and co-infections are common in endemic communities^4^. Transmission is closely linked to poverty, inadequate sanitation, and limited access to safe water^5^. School-aged children carry a particularly heavy burden: chronic infection during childhood contributes to anaemia, growth retardation, impaired cognitive development, and reduced school performance, with consequences that can persist into adulthood^3,5^. In Nigeria, prevalence varies markedly across regions, with children in rural communities most affected^6,7^.

Current control efforts in Nigeria and across sub-Saharan Africa rely primarily on preventive chemotherapy through mass drug administration (MDA), following WHO guidelines^8^. These programmes have succeeded in reducing the prevalence of heavy-intensity infections in many settings. Yet evidence suggests that treatment-only strategies provide temporary relief and have limited impact on transmission dynamics where re-infection rates remain high and environmental contamination persists^8,9^. In Plateau State the setting for this study MDA campaigns have been implemented intermittently, but transmission of both schistosomiasis and STH continues, particularly in communities with poor water, sanitation, and hygiene (WASH) infrastructure^6^.

The shift toward elimination as a programmatic goal has drawn attention to a critical limitation of current surveillance approaches. The diagnostic methods recommended by WHO Kato-Katz thick smear for intestinal helminths and *S. mansoni*, and urine filtration for *S. haematobium* are inexpensive and widely deployed, but their sensitivity is limited^9,10^. These techniques depend on the presence of parasite eggs in the specimen, and their accuracy declines markedly in low-intensity infections, where egg output may be intermittent and scanty^11,12^. Day-to-day variation in egg excretion, technician expertise, and the volume of specimen examined further contribute to diagnostic uncertainty. A proportion of infected individuals those harbouring pre-patent infections, very low worm burdens, or infections with irregular egg shedding may consequently be classified as uninfected by microscopy. Such sub-microscopic infections, while individually representing low parasite loads, could collectively sustain transmission and undermine elimination targets if they go unrecognised^9,13^.

Molecular diagnostic methods, including polymerase chain reaction (PCR) and quantitative real-time PCR (qPCR), offer substantially greater analytical sensitivity than conventional microscopy, enabling detection of parasite DNA in specimens where eggs are absent or too few to be identified microscopically^14,15^. Several studies in diverse endemic settings have reported that molecular techniques detect additional infections missed by microscopy, though the magnitude and programmatic significance of this difference remain incompletely characterised in many regions^14,16^.

This study aimed to compare the detection rates of conventional microscopy with probe-based multiplex real-time PCR and quantitative real-time PCR for schistosomiasis and soil-transmitted helminths among primary school children in Plateau State, Nigeria, and to estimate the proportion of sub-microscopic infections missed by standard diagnostic methods. The findings are intended to inform discussions on the role of molecular diagnostics in elimination programme surveillance.

## Methods

### Study setting and population

We conducted this cross-sectional study in Plateau State, central Nigeria, across six Local Government Areas (LGAs): Mangu, Jos North, Jos South, Barkin Ladi, Riyom, and Bokkos. Plateau State lies on the Jos Plateau at an elevation of approximately 1,200 m above sea level and experiences a sub-humid tropical climate with distinct wet (April–October) and dry (November–March) seasons. Annual rainfall ranges from approximately 1,200 mm in the northern LGAs (Jos North, Jos South) to over 1,400 mm in the southern areas (Bokkos, Riyom). The state is drained by several rivers and streams—including tributaries of the Benue River system that provide water for domestic use, agriculture, and recreation, creating conditions that support schistosome transmission. Subsistence farming and animal husbandry are the primary occupations, and many communities depend on untreated surface water. WASH infrastructure is limited in rural areas, with open defecation remaining common. These ecological and socioeconomic conditions sustain transmission of both schistosomiasis and soil-transmitted helminthiasis^6,7^.

### Sample size calculation

The minimum sample size was calculated using the standard formula for cross-sectional studies: n = (Z²α × P × (1 − P))/d², where Zα = 1.96 (95% confidence level), *P* = 0.15, and d = 0.02 (desired precision). The expected prevalence of 15% was based on pooled estimates of combined schistosomiasis and STH prevalence from prior surveys in Plateau State and neighbouring states^6,7^. This yielded a minimum required sample of 1,225 participants. Accounting for a 10% non-response rate, our target was 1,348. The final enrolled sample of 1,368 exceeded this requirement.

## Study design and participants

We employed systematic random sampling to recruit 1,368 children from six primary schools (one per LGA), selecting every third eligible child from school registers until reaching target sample sizes proportional to school enrolment. All participants were enrolled in primary school at the time of the study. In rural Nigeria, late school enrolment and grade repetition are common, which accounts for the inclusion of children up to 19 years of age within the primary school setting^17^.

Inclusion criteria: Children aged 5–19 years attending participating primary schools, with written informed consent from a parent or legal guardian.

Exclusion criteria: Children outside the specified age range, those with severe illness precluding participation, or those absent during data collection.

### Sample collection

Biological specimens were collected between 10:00 AM and 2:00 PM to optimise diagnostic sensitivity^10^. Each participant provided a mid-stream urine sample (25 mL) and a fresh stool sample (approximately 2 g) in sterile, labelled containers. Specimens were transported to the designated laboratory within two hours under cold chain conditions. A total of 1,332 samples were successfully processed (97.4% completion rate), with 36 excluded owing to storage failure or insufficient volume.

### Data provenance and related publications

The Plateau State dataset analysed in this study was subsequently incorporated into a broader multi-state investigation examining helminth prevalence across six Nigerian states (Asaga et al., *Acta Tropica*, doi: 10.1016/j.actatropica.2026.108039). The present manuscript was submitted prior to that multi-state analysis and focuses specifically on the diagnostic comparison between microscopy and molecular methods at the single-state level, which was not the primary analytical objective of the broader study.

### Microscopic analysis

For *S. haematobium* detection, urine samples were processed using the standard filtration technique with Nucleopore membrane filters (13 mm diameter, 12 μm pore size). Ten millilitres of each specimen were filtered, mounted on microscope slides, stained with Lugol’s iodine, and examined at 100× and 400× magnifications^11^. For *S. mansoni* and soil-transmitted helminths (*A. lumbricoides*,* T. trichiura*,* N. americanus*,* and A. duodenale*), stool samples were processed using the Kato-Katz technique^12^. Duplicate thick smears were prepared from each specimen using standardised templates, and glycerol-soaked cellophane coverslips were applied. Egg counts were expressed as eggs per gram of stool (EPG). All microscopic examinations were performed independently by two qualified laboratory technicians with more than five years of parasitological experience. Discordant results were resolved by re-examination by a senior parasitologist.

### Molecular analysis

A stratified random subset of 585 samples (42.8% of the total cohort) was selected for molecular analysis. Stratification ensured proportional representation by study site, sex, age group, and microscopy result (both positive and negative specimens were included). This design allowed estimation of both the sensitivity gain of molecular methods over microscopy and the proportion of sub-microscopic infections in the study population.

DNA was extracted from stool and urine specimens using the QIAamp DNA Stool Mini Kit and QIAamp DNA Mini Kit respectively (Qiagen, Hilden, Germany), following the manufacturer’s protocols with minor modifications (an additional bead-beating step was included to improve lysis of helminth eggs). DNA concentration and purity were assessed by spectrophotometry (NanoDrop, Thermo Fisher Scientific).

#### Probe-based multiplex real-time PCR

We performed probe-based multiplex real-time PCR for simultaneous detection and species identification of *S. mansoni*,* S. haematobium*,* N. americanus*,* A. duodenale*,* A. lumbricoides*, and *T. trichiura* using species-specific hydrolysis (TaqMan) probes labelled with distinct fluorophores (FAM, HEX, Cy5, Texas Red), following established protocols^14,18,19^. Amplification was performed in 25 µL reactions containing 12.5 µL of 2× TaqMan Multiplex Master Mix (Applied Biosystems), species-specific forward and reverse primers at 300 nM each, species-specific probes at 200 nM each, 2 µL of DNA template, and nuclease-free water to final volume. Thermal cycling comprised: 50 °C for 2 min (UNG activation), 95 °C for 10 min (polymerase activation), followed by 40 cycles of 95 °C for 15 s and 60 °C for 60 s. Species identification was determined by the fluorescence channel in which amplification was detected; no gel electrophoresis was required. Reactions were run on a LightCycler 96 system (Roche, Switzerland). For quality assurance, a subset of discordant samples (*n* = 15) underwent Sanger sequencing for confirmatory species verification.

#### Probe-based quantitative real-time PCR (qPCR)

Species-specific quantification was performed using probe-based singleplex qPCR assays, run separately for each species to avoid competitive amplification effects that can compromise quantitative accuracy in multiplexed reactions. Target genomic regions were: internal transcribed spacer 2 (ITS2) for *Schistosoma species*, ITS1 for hookworm species, and mitochondrial cytochrome oxidase I (cox1) for *A. lumbricoides* and *T. trichiura*^14,19^. Reactions were prepared in 20 µL volumes: 10 µL 2× TaqMan Universal Master Mix (Applied Biosystems), species-specific forward and reverse primers at 300 nM, species-specific hydrolysis probe at 200 nM, 2 µL template DNA, and nuclease-free water to final volume. Thermal cycling conditions were: 50 °C for 2 min, 95 °C for 10 min, followed by 45 cycles of 95 °C for 15 s and 60 °C for 60 s. Standard curves were generated from 10-fold serial dilutions of quantified genomic DNA extracted from known numbers of parasite eggs (10⁴ to 10⁰ egg equivalents). Amplification efficiency ranged from 92% to 98%, with R² values of 0.991–0.998 across all species (Supplementary Figure S1). Cycle threshold (Ct) values were converted to egg genome equivalents using these standard curves. Samples with Ct values < 40 and characteristic amplification curves were classified as positive. All assays were pre-validated using genomic DNA from verified parasite specimens.

### Contamination prevention and cross-reactivity controls

DNA extraction, PCR setup, and post-PCR analysis were performed in physically separated areas with dedicated equipment. UV decontamination of work surfaces and equipment was conducted before and after each session. Positive controls (genomic DNA from verified parasite specimens), negative extraction controls, and no-template controls (nuclease-free water) were included in every run. Primer and probe specificity was confirmed during assay optimisation by testing against non-target parasite DNA to exclude cross-reactivity.

#### Quality assurance and validation

All qPCR reactions were performed in duplicate as standard practice. Additionally, 10% of samples were re-extracted from original specimens and re-tested to assess inter-assay reproducibility, yielding 97% raw agreement (κ = 0.94, 95% CI: 0.89–0.99). External quality control was maintained through participation in international proficiency testing programmes.

#### Risk factor data collection

A pre-tested, structured questionnaire was administered to the caregivers of participating children to collect information on household water source, sanitation facilities, frequency of water contact activities, and sociodemographic variables. The questionnaire was pilot-tested in a non-study school and revised for clarity prior to field deployment.

### Statistical analysis

Data were analysed using SPSS version 21.0 (IBM Corporation, Armonk, NY), with statistical significance set at *p* < 0.05. Descriptive statistics were used to characterise the study population. We calculated 95% confidence intervals for all prevalence estimates using the Wilson score method. McNemar’s test compared paired detection rates between microscopy and molecular methods, with exact p-values calculated for small sample sizes. Sensitivity, specificity, positive predictive value, and negative predictive value of microscopy were calculated relative to molecular methods as the reference standard.

For risk factor analysis, variables associated with infection at *p* < 0.20 in univariable logistic regression were entered into a multivariable model. Multicollinearity was assessed using variance inflation factors (VIF < 5 threshold). Influential observations were evaluated by Cook’s distance. Results are reported as adjusted odds ratios (AOR) with 95% confidence intervals. Model fit was assessed using the Hosmer-Lemeshow goodness-of-fit test. Spearman’s rank correlation assessed the relationship between qPCR Ct values and microscopic egg counts among dual-positive specimens.

### Ethical considerations

All methods were performed in accordance with the Declaration of Helsinki and WHO guidelines for parasitological surveys^20^. Ethical approval was obtained from the Plateau State Ministry of Health Ethics Committee and the University of Jos Institutional Review Board (Reference: NO/2024/076). Written informed consent was obtained from parents or legal guardians of all participants. Children aged seven years and above provided additional assent. All individuals found positive received appropriate treatment according to national guidelines.

## Results

### Study population characteristics

The study enrolled 1,368 participants: 724 males (52.9%) and 644 females (47.1%), aged 5–19 years (mean age: 10.8 ± 2.4 years). The age distribution comprised 673 children aged 5–9 years (49.2%), 507 aged 10–14 years (37.1%), and 188 aged 15–19 years (13.7%). A total of 1,332 samples were successfully processed (97.4% completion rate).

### Parasitological results by microscopy

Based on microscopy, the overall prevalence of parasitic infection was 20.7% (95% CI: 18.6–22.9%). This comprised 279 single-species infections and 4 co-infections (0.3%); the co-infections involved concurrent schistosomiasis and STH (S. mansoni with hookworm in three cases and S. haematobium with A. lumbricoides in one). Overall schistosomiasis prevalence was 7.0% (95% CI: 5.7–8.6%), while STH prevalence was 14.0% (95% CI: 12.2–15.9%).

Species-specific prevalence was as follows: *S. mansoni* 4.9% (95% CI: 3.9–6.1%), *S. haematobium* 2.1% (95% CI: 1.4–2.9%), hookworm 9.6% (95% CI: 8.1–11.3%), *A. lumbricoides* 5.9% (95% CI: 4.7–7.3%), and *T. trichiura* 0.4% (95% CI: 0.2–0.8%).

### Molecular detection results

**Fig. 1:**
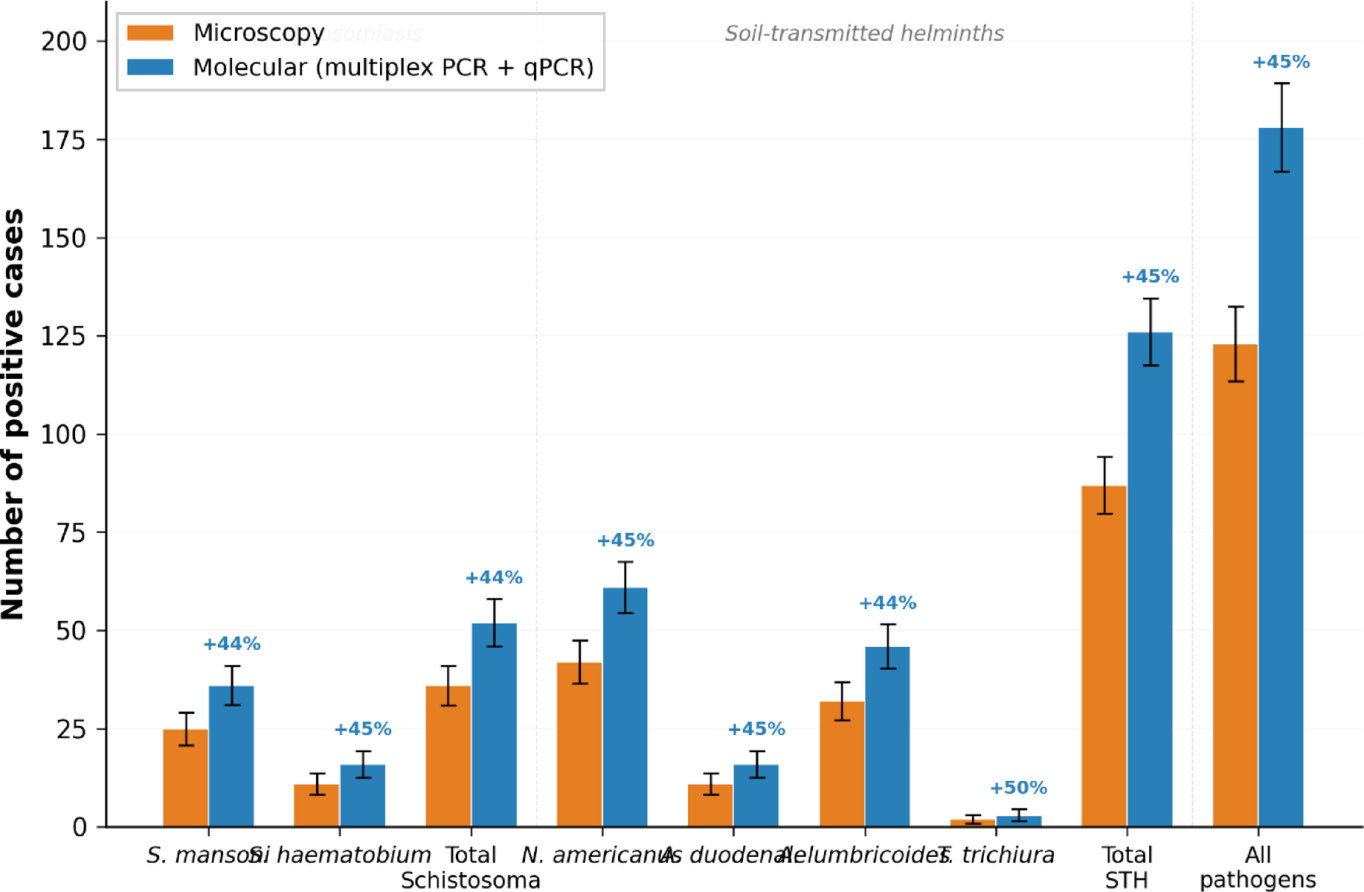
Number of positive cases detected by microscopy and molecular methods (probe-based multiplex real-time PCR and qPCR) for each parasite species, with 95% confidence intervals (*n* = 585). Percentage increases in detection by molecular methods are indicated above the bars.

Table 1 presents the comparative detection between microscopy and molecular methods among the 585 samples analysed by both techniques. Molecular methods detected 178 positive cases compared with 123 by microscopy, representing a 44.7% increase in detection (95% CI: 36.2–53.8%, *p* < 0.001). Fifty-five specimens (9.4%, 95% CI: 7.1–12.1%) were molecular-positive but microscopy-negative (Fig. 1). All microscopy-positive specimens were confirmed positive by molecular methods.

**Table 1:** Diagnostic comparison between microscopy and molecular methods (*n* = 585).

Panel A. Overall concordance between microscopy and molecular methods			
	Molecular +	Molecular −	Total
Microscopy +	123	0	123
Microscopy −	55	407	462
Total	178	407	585

Panel B. Species-specific detection comparison					
Pathogen	Microscopy positive	Molecular positive	Molecular +, microscopy −	Increase (%, 95% CI)	*p*-value
*S. mansoni*	25	36	11	44.0 (28.5–61.2)	< 0.01
*S. haematobium*	11	16	5	45.5 (21.3–74.8)	< 0.05
Total Schistosoma	36	52	16	44.4 (28.1–63.2)	< 0.001
*N. americanus*	42	61	19	45.2 (32.1–59.7)	< 0.001
*A. duodenale*	11	16	5	45.5 (21.3–74.8)	< 0.05
*A. lumbricoides*	32	46	14	43.8 (26.8–63.1)	< 0.01
*T. trichiura*	2	3	1	50.0 (6.8–93.2)	0.32
Total STH	87	126	39	44.8 (34.9–55.7)	< 0.001
All pathogens	123	178	55	44.7 (36.2–53.8)	< 0.001

McNemar’s test: χ² = 55.0, p < 0.001. Concordance: 90.6%. Sensitivity of microscopy (molecular as reference): 69.1%. p-values calculated using McNemar’s test for paired proportions.

### Geographic distribution of infections

Table 2 shows the distribution of infections by microscopy and molecular methods across the six LGAs. Microscopy-based prevalence ranged from 19.2% in Barkin Ladi to 22.5% in Jos North. Among molecularly analysed samples, the proportion of sub-microscopic infections (molecular-positive, microscopy-negative) ranged from 7.2% in Riyom to 12.1% in Jos North.

**Table 2:** Geographic distribution of infections by local government area.

LGA	Total samples	Microscopy positive (%, 95% CI)	Molecular subset (*n*)	Submicroscopic infections† (%, 95% CI)
Mangu	228	45 (19.7, 14.8–25.5)	98	8 (8.2, 3.6–15.5)
Jos North	231	52 (22.5, 17.3–28.4)	99	12 (12.1, 6.4–20.6)
Jos South	225	48 (21.3, 16.2–27.3)	96	9 (9.4, 4.4–17.1)
Barkin Ladi	229	44 (19.2, 14.3–25.0)	98	10 (10.2, 5.0–18.1)
Riyom	227	46 (20.3, 15.2–26.2)	97	7 (7.2, 2.9–14.4)
Bokkos	228	48 (21.1, 15.9–27.1)	97	9 (9.3, 4.3–16.8)

†Submicroscopic infections: molecular-positive, microscopy-negative specimens within the molecular subset.

### Infection intensity distribution

Table 3 shows the infection intensity classification among microscopy-positive cases according to WHO thresholds^21^. For *S. haematobium*, 86.2% of infections were light-intensity (< 50 eggs/10 mL) and 13.8% were heavy-intensity (≥ 50 eggs/10 mL). For *S. mansoni*, 88.1% were light (< 100 EPG) and 3.0% were heavy (≥ 400 EPG). Among STH species, the majority of infections fell within the light-intensity category (89.3–100%).

**Table 3:** Infection intensity distribution among microscopy-positive cases, classified according to WHO thresholds.

Pathogen (*n*)	Light intensity *n* (%, 95% CI)	Moderate intensity *n* (%, 95% CI)	Heavy intensity *n* (%, 95% CI)	Geometric mean (95% CI)
*S. haematobium* (*n* = 29)	25 (86.2, 68.3–96.1)	–	4 (13.8, 3.9–31.7)	18.7 eggs/10 mL (12.4–28.2)
*S. mansoni* (*n* = 67)	65 (97.0, 89.6–99.6)	–	2 (3.0, 0.4–10.4)	32.4 EPG (24.8–42.3)
Hookworm (*n* = 131)	117 (89.3, 82.8–94.0)	12 (9.2, 4.8–15.8)	2 (1.5, 0.2–5.4)	312.8 EPG (267.3–365.9)
*A. lumbricoides* (*n* = 81)	74 (91.4, 83.0–96.5)	6 (7.4, 2.8–15.4)	1 (1.2, 0.0–6.6)	1,247 EPG (956–1,627)
*T. trichiura* (*n* = 6)	6 (100.0, 54.1–100.0)	0 (0.0)	0 (0.0)	24.5 EPG (8.7–69.1)

WHO intensity thresholds: S. haematobium: light < 50, heavy ≥ 50 eggs/10 mL; S. mansoni: light 1–99, heavy ≥ 400 EPG (no moderate category for schistosomiasis per WHO classification). STH moderate/heavy thresholds per WHO guidelines^21^. — = category not applicable per WHO classification.

### Demographic distribution of infections

Tables 4 and 5 present sex-specific and age-stratified prevalence data respectively. Overall infection prevalence did not differ significantly between males (21.4%, 95% CI: 18.4–24.6%) and females (19.9%, 95% CI: 16.9–23.2%; *p* = 0.568). Males had significantly higher *S. mansoni* prevalence than females (6.1% vs. 3.6%, crude OR = 1.7, 95% CI: 1.0–2.9, *p* = 0.043). Schistosomiasis prevalence peaked in the 10–14-year age group (9.5%), whilst STH prevalence was highest among 5–9-year-olds (16.9%) and declined with age (*p* = 0.001) (Table 5).

**Table 4:** Sex-specific prevalence of parasitic infections by microscopy.

Infection	Males (*n* = 724) n (%, 95% CI)	Females (*n* = 644) n (%, 95% CI)	Crude OR (95% CI)	*p*-value
Total infections	155 (21.4, 18.4–24.6)	128 (19.9, 16.9–23.2)	1.1 (0.8–1.4)	0.568
Schistosomiasis	58 (8.0, 6.1–10.3)	38 (5.9, 4.2–8.1)	1.4 (0.9–2.1)	0.126
S. mansoni	44 (6.1, 4.4–8.1)	23 (3.6, 2.3–5.3)	1.7 (1.0–2.9)	0.043*
S. haematobium	14 (1.9, 1.1–3.2)	15 (2.3, 1.3–3.8)	0.8 (0.4–1.7)	0.617
STH infections	112 (15.5, 12.9–18.4)	79 (12.3, 9.8–15.2)	1.3 (1.0–1.8)	0.087

OR = crude odds ratio. *Statistically significant at *p* < 0.05.

**Table 5:** Age-specific prevalence of parasitic infections by microscopy.

Infection	5–9 years (*n* = 673) n (%, 95% CI)	10–14 years (*n* = 507) n (%, 95% CI)	15–19 years (*n* = 188) n (%, 95% CI)	*p*-value†
Total infections	145 (21.5, 18.5–24.8)	111 (21.9, 18.4–25.7)	27 (14.4, 9.7–20.3)	0.043
Schistosomiasis	35 (5.2, 3.7–7.2)	48 (9.5, 7.1–12.4)	13 (6.9, 3.7–11.7)	0.005
STH infections	114 (16.9, 14.2–20.0)	63 (12.4, 9.7–15.7)	14 (7.4, 4.1–12.2)	0.001

†p-value from chi-squared test for trend across age groups.

### Environmental and WASH risk factors

**Fig. 2:**
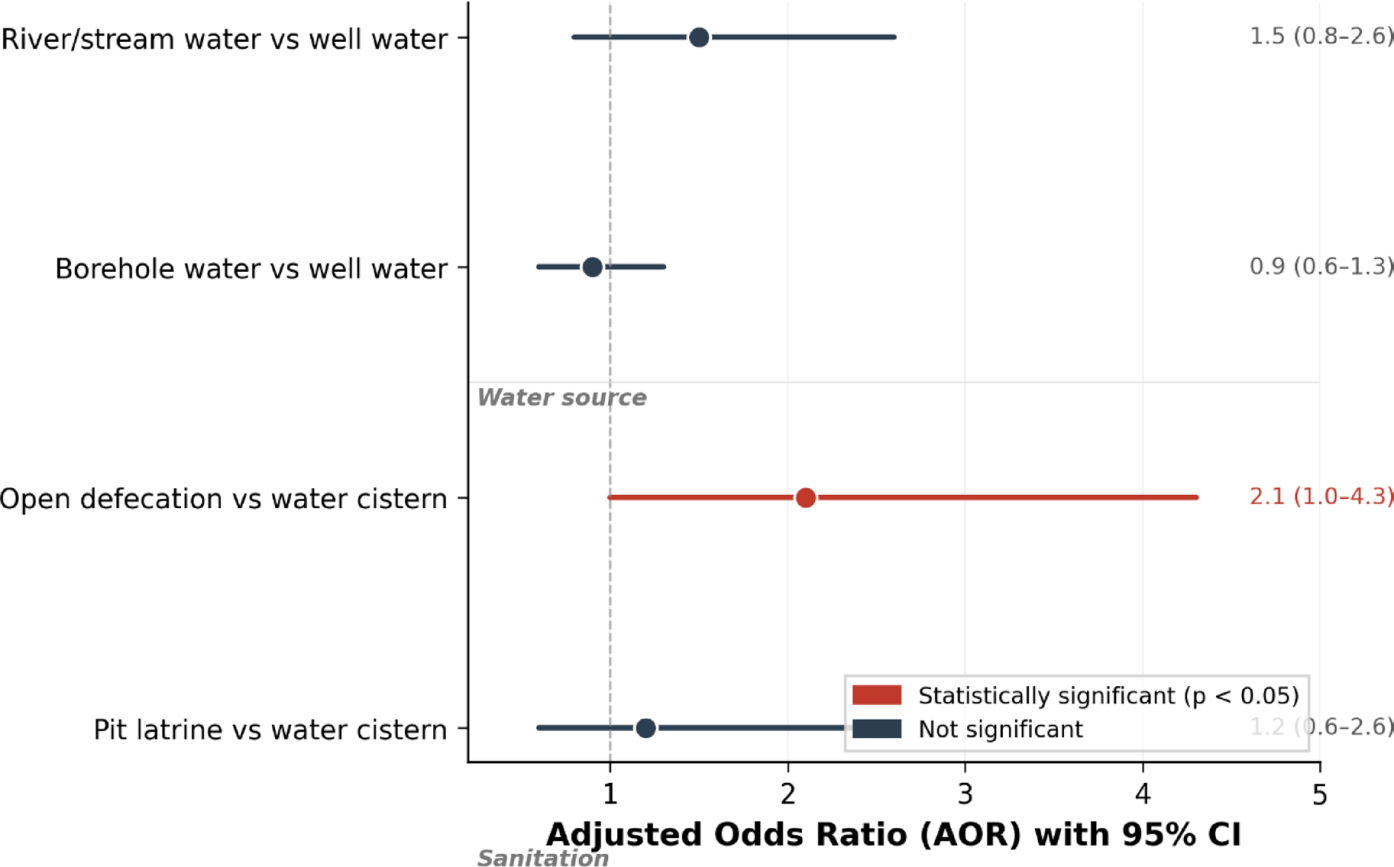
Forest plot of adjusted odds ratios (AOR) and 95% confidence intervals for the association between water source, sanitation practices, and overall parasitic infection. The dashed line indicates AOR = 1 (null). Red markers indicate statistically significant associations (*p* < 0.05).

Table 6; Fig. 2 present the associations between water source, sanitation practices, and infection prevalence. In multivariable analysis, open defecation was significantly associated with increased odds of infection compared with water cistern use (AOR = 2.1, 95% CI: 1.0–4.3, *p* = 0.047). River or stream water use was associated with elevated but non-significant odds of infection compared with well water (AOR = 1.5, 95% CI: 0.8–2.6, *p* = 0.176). Frequent water contact activities were independently associated with infection (AOR = 1.6, 95% CI: 1.2–2.1, *p* = 0.001).

**Table 6:** Association between water source, sanitation practices, and parasitic infection prevalence.

Risk factor	Category	*n*	Infections *n* (%, 95% CI)	AOR† (95% CI)	*p*-value
Water source	Well water	1,094	225 (20.6, 18.2–23.1)	Reference	
	Borehole	205	39 (19.0, 13.9–25.2)	0.9 (0.6–1.3)	0.572
	River/stream	69	19 (27.5, 17.6–39.1)	1.5 (0.8–2.6)	0.176
Sanitation	Water cistern	55	8 (14.5, 6.5–27.2)	Reference	—
	Pit latrine	721	125 (17.3, 14.6–20.3)	1.2 (0.6–2.6)	0.597
	Open defecation	592	150 (25.3, 21.9–29.0)	2.1 (1.0–4.3)	0.047*

†Adjusted for age, sex, water contact frequency, and clustering by school. *Statistically significant at *p* < 0.05. — = reference category.

#### Correlation between qPCR Ct values and egg counts

**Fig. 3:**
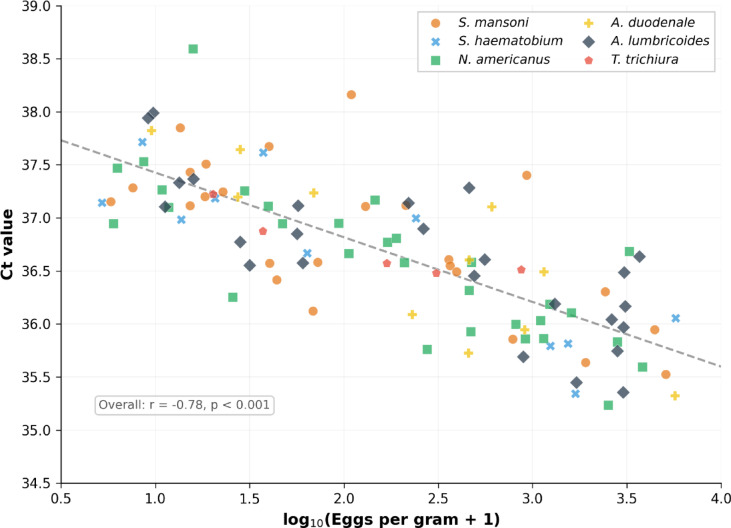
Correlation between qPCR cycle threshold (Ct) values and log-transformed egg counts (EPG or eggs/10 mL urine) for six parasite species among specimens positive by both microscopy and qPCR. Each point represents an individual specimen. The dashed line shows the overall linear regression.

Among specimens positive by both microscopy and qPCR, Ct values ranged from 35.0 to 38.7 (Fig. 3). An inverse correlation was observed between Ct values and log-transformed egg counts across all species (Spearman *r* = − 0.65 to − 0.82, *p* < 0.001). The relatively high Ct values are consistent with the predominance of low-intensity infections in this population. Individual Ct values for all molecular-positive specimens are provided in Supplementary Table S1.

## Discussion

In this diagnostic comparison study, probe-based molecular methods detected 44.7% more parasitic infections than conventional microscopy among primary school children in Plateau State, Nigeria. Nearly one in ten specimens negative by microscopy yielded positive results by molecular testing. These findings add to a growing body of evidence that standard parasitological methods while practical and widely deployed may substantially underestimate infection prevalence, particularly in settings where low-intensity infections predominate^9,14,16^. A broader multi-state investigation including all six Nigerian states, and incorporating the Plateau State data analysed here, has now been published in *Acta Tropica*^22^, providing national-level context for the diagnostic patterns observed in this single-state analysis.

The magnitude of the detection difference (44.7%, 95% CI: 36.2–53.8%) was consistent across both schistosomiasis and STH species, suggesting a systematic limitation of egg-based microscopy rather than a species-specific artefact. This is broadly in line with findings from other endemic settings. Studies in Zanzibar, Côte d’Ivoire, and Cameroon have reported 30–60% additional infections detected by molecular methods relative to microscopy, though direct comparisons are complicated by differences in study design, molecular platforms, and target populations^9,14,23^. The relatively uniform increase across species in our data may reflect the study setting a moderate-endemicity area where most detectable infections already fall within the light-intensity range.

It is worth emphasising what these data can and cannot demonstrate. The additional infections detected by molecular methods were, by definition, below the threshold of microscopic detection. We describe these as sub-microscopic rather than “cryptic” infections, since the latter term carries specific epidemiological connotations relating to transmission dynamics in near-elimination settings that our cross-sectional data cannot substantiate^24^. Possible explanations for the microscopy-negative, PCR-positive results include pre-patent infections, very low worm burdens with intermittent or absent egg production, and the inherent sampling limitations of single stool or urine specimens^11,12^. A single Kato-Katz examination, in particular, is known to miss a considerable proportion of light-intensity infections^12,25^.

The Ct values observed ranged from 35.0 to 38.7, which are high and warrant careful interpretation. Such values are consistent with very low parasite DNA concentrations and, correspondingly, low worm burdens. Whilst the standard curves showed acceptable efficiency (92–98%) and linearity (R² = 0.991–0.998), results at the upper end of the Ct range carry greater analytical uncertainty. The use of TaqMan probe-based chemistry provides an important specificity advantage at high Ct values compared with intercalating dye approaches, since positive results require both amplification and specific probe hybridisation. Nonetheless, the possibility of false positives at the extreme detection limit cannot be entirely excluded, and future studies might benefit from additional confirmatory steps such as sequencing of borderline-positive samples^14^.

The geographic distribution of infections was relatively uniform across the six LGAs (19.2–22.5% by microscopy), likely reflecting broadly similar ecological conditions and WASH infrastructure across the study sites. The proportion of sub-microscopic infections showed somewhat greater variation (7.2–12.1%), with Jos North showing the highest rate possibly related to its more urban and periurban character, where lower overall transmission intensity could paradoxically yield a higher proportion of low-intensity infections. This finding should be interpreted cautiously given the modest sample sizes at the LGA level.

The low prevalence of *T. trichiura* (0.4%) is consistent with the semi-arid ecological conditions of Plateau State, which are generally less conducive to transmission of this moisture-dependent species^4,26^. The predominance of *N. americanus* among molecularly identified hookworm infections (78.3%) aligns with established epidemiological patterns in sub-Saharan Africa^27^.

Open defecation emerged as the strongest modifiable risk factor (AOR = 2.1, *p* = 0.047), underscoring the importance of sanitation improvements alongside chemotherapy-based interventions. The association between male sex and *S. mansoni* infection (OR = 1.7, *p* = 0.043) likely reflects gendered patterns of water contact in rural Nigerian communities, where boys are more commonly involved in fishing, swimming, and agricultural activities near water bodies^17,28^.

These findings have potential implications for elimination programme design, though we are cautious not to overstate them on the basis of a single cross-sectional study. If microscopy consistently misses a substantial fraction of infections, then prevalence thresholds based solely on microscopic detection could lead to premature conclusions about control or elimination progress. Whether sub-microscopic infections contribute meaningfully to ongoing transmission a critical question for programme decisions cannot be answered by our data and requires longitudinal studies incorporating both parasitological and transmission-based endpoints^24,29^.

### Limitations

Several limitations should be considered. First, the cross-sectional design does not allow assessment of temporal trends or the longitudinal significance of sub-microscopic infections. Second, a single specimen was collected per participant; repeated sampling would likely have increased the sensitivity of microscopy and narrowed the gap between methods^12,25^. Third, we did not collect information on participants’ treatment history, which may have influenced current infection status and the relationship between detection methods. Fourth, the molecular subset represented 42.8% of the total cohort; although stratified sampling was used, extrapolation to the entire study population requires caution. Fifth, the high Ct values observed (35–38.7) raise questions about the proportion of true versus false positives at the lower limits of detection, despite the inherent specificity advantages of TaqMan probe chemistry and the inclusion of appropriate controls. Finally, this study was conducted in a moderate-endemicity setting; the extent to which these findings generalise to low- or high-endemicity areas remains uncertain.

## Conclusion

Molecular methods detected 44.7% more parasitic infections than conventional microscopy in this population of primary school children, indicating that a substantial proportion of infections went undetected by standard diagnostic methods. These findings suggest that microscopy-based surveillance may underestimate the true infection burden, particularly in settings where low-intensity infections predominate. The integration of molecular diagnostics into surveillance frameworks could improve the accuracy of elimination assessments, though operational considerations and the epidemiological significance of sub-microscopic infections require further investigation. Sustained improvements in water, sanitation, and hygiene infrastructure—particularly addressing open defecation—remain essential alongside diagnostic advances for achieving and maintaining control of schistosomiasis and soil-transmitted helminthiasis.

## Supplementary Information

Below is the link to the electronic supplementary material.

Supplementary Material 1

Supplementary Material 2

Supplementary Material 3

Supplementary Material 4

## Data Availability

The datasets analysed during this study are available from the corresponding author on reasonable request. Supplementary data (standard curves, individual Ct values) are provided as supplementary files.
