# Supplementary figures and images for: Sub-microscopic schistosomiasis and soil-transmitted helminths in school children: molecular diagnostic evidence and implications for disease elimination
Source: Sci Rep. 2026 Mar 18;16:9236. doi: 10.1038/s41598-026-44877-8 (PMC13000292; doi:10.1038/s41598-026-44877-8)

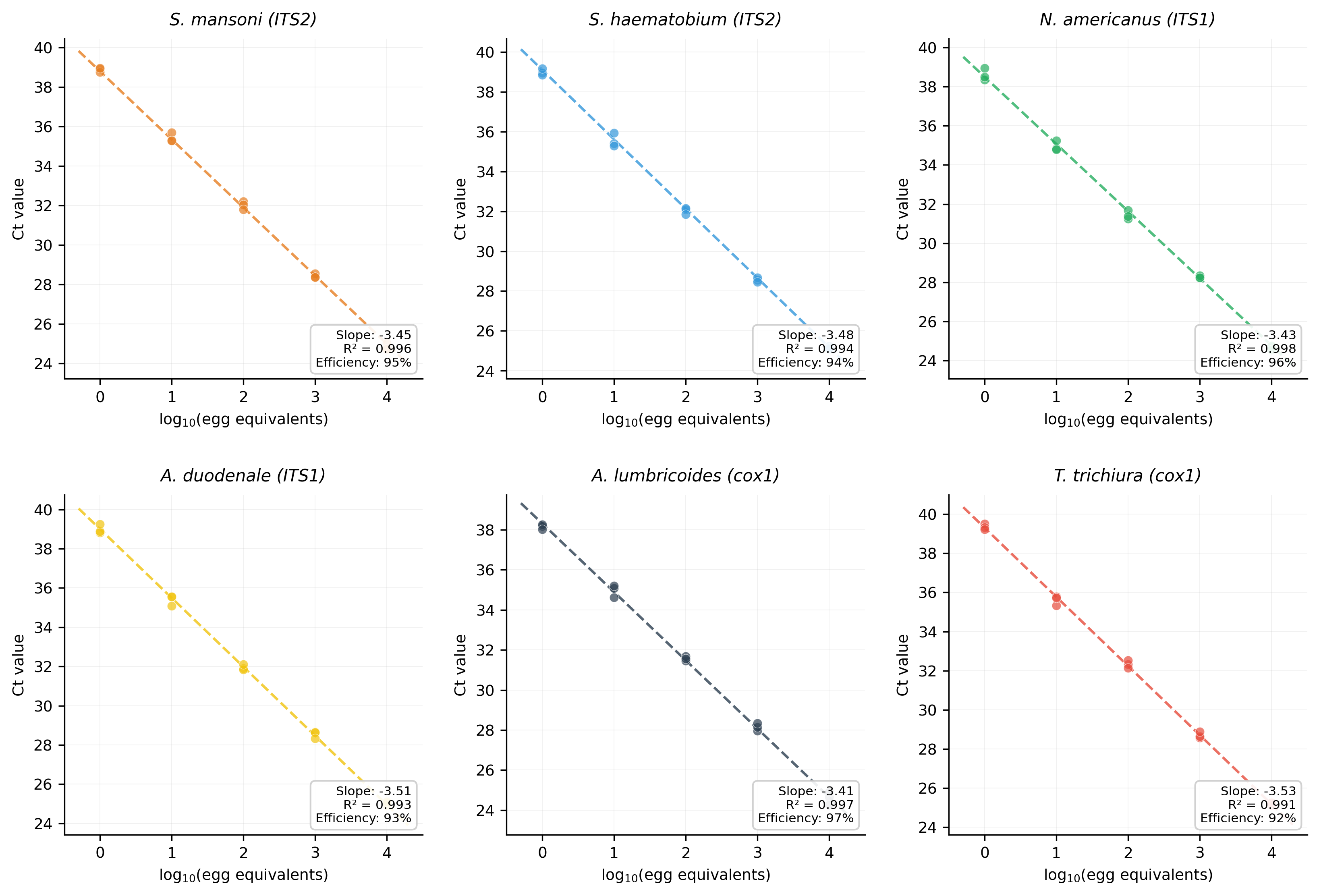

Supplement: Supplementary file 1 — Supplementary Material 1 [file 41598_2026_44877_MOESM1_ESM.png]

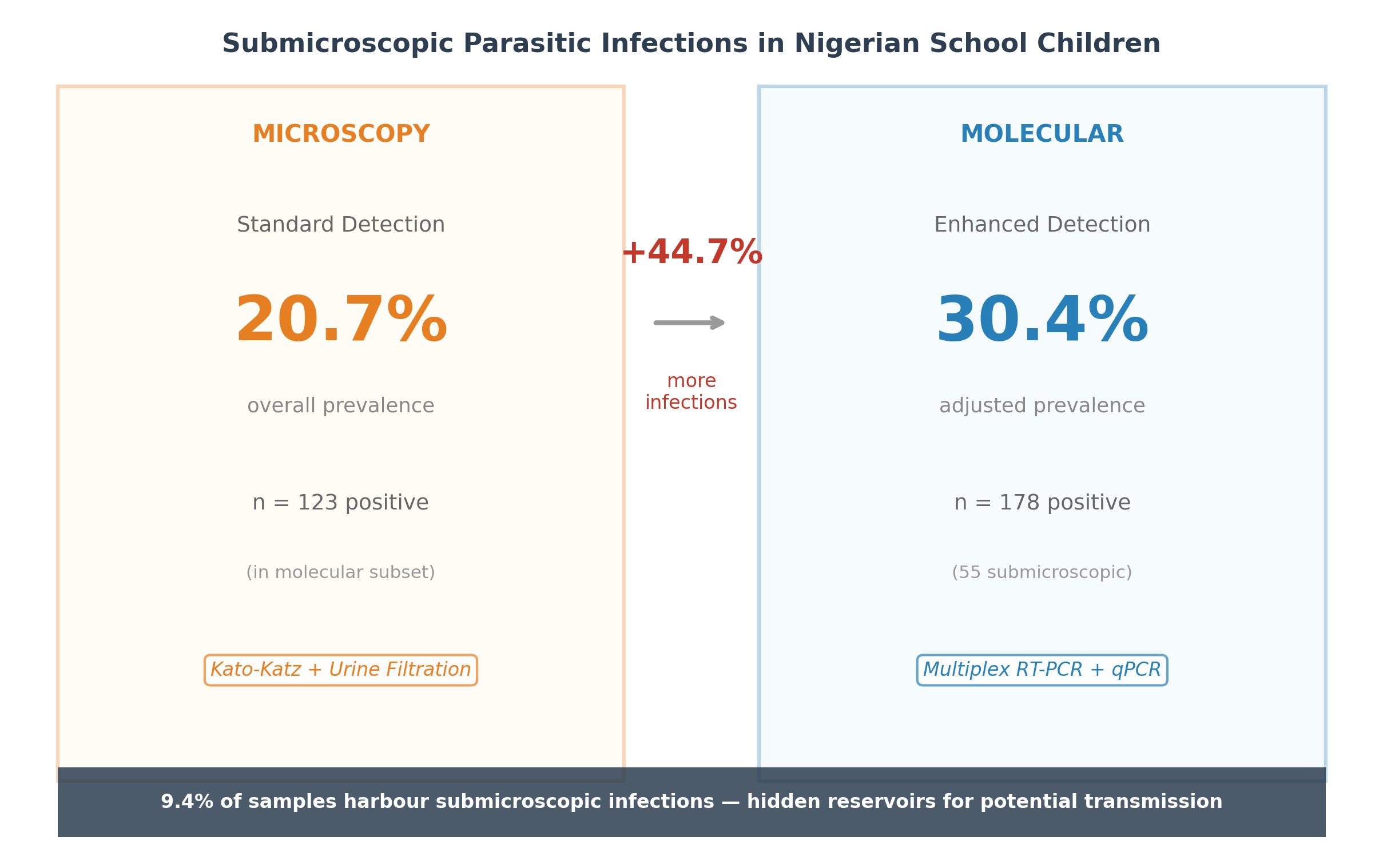

Supplement: Supplementary file 2 — Supplementary Material 2 [file 41598_2026_44877_MOESM2_ESM.png]
