# Supplementary material for: Sub-microscopic schistosomiasis and soil-transmitted helminths in school children: molecular diagnostic evidence and implications for disease elimination
Source: Sci Rep. 2026 Mar 18;16:9236. doi: 10.1038/s41598-026-44877-8 (PMC13000292; doi:10.1038/s41598-026-44877-8)
Supplement: Supplementary file 4 — Supplementary Material 4 [file 41598_2026_44877_MOESM4_ESM.docx]

**Figure Graphical Abstract.** Submicroscopic parasitic infections in Nigerian school children: molecular diagnostics detected 44.7% more infections than conventional microscopy, revealing that 9.4% of specimens harboured submicroscopic infections undetected by standard methods.
